# The changes of oral health-related quality of life and satisfaction after surgery-first orthognathic approach: a longitudinal prospective study

**DOI:** 10.1186/s13005-015-0098-1

**Published:** 2016-01-05

**Authors:** Shengbin Huang, Weiting Chen, Zhenyu Ni, Yu Zhou

**Affiliations:** Department of Prosthodontics, Hospital of Stomatology, Wenzhou Medical University, Wenzhou, China; Department of Orthodontics, Hospital of Stomatology, Wenzhou Medical University, 113 West College Road, 325000 Wenzhou, China

**Keywords:** Quality of life, Orthognathic surgery, Surgery-first, Orthodontic-first

## Abstract

**Background:**

To our best knowledge, there was little research to assess the changes of quality of life and satisfaction after orthognathic in one trial. The aim of this study was to evaluate the changes of oral health related quality of life and satisfaction between surgery-first and orthodontic-first orthognathic surgery.

**Methods:**

Fifty Chinese orthognathic adluts patients completed two questionnaires: the Dental Impact on Daily Living questionnaire for assessment of his/her satisfaction and 14-item Oral Health Impact Profile for assessment of patient’s quality of life. The subjects completed six sets of interviews and clinical evaluations at before treatment; 1 month after surgery (surgery-first); 6 months after treatment; 12 months after treatment ; and 18 month after treatment ; the finished treatment. The pre and post surgical orthodontic period was also recorded. Chi square tests and repeated-measures analysis of variance (ANOVA) were used to compare categorical variables and measure results. All analyses were carried out used Stata software.

**Results:**

The quality of life was significant improved when finished treatment and the amounts of change did not show any significant difference in each domain and at 1, 6, 12 month after orthognathic surgery between two groups. However, in orthodontic-first group, the quality of life was deteriorated before orthognathic surgery. In surgery-first group, the quality of life was immediately improved which lead to better satisfaction.

**Conclusions:**

Although the quality of life scores was no significant difference between two groups, surgery-first treatment could significant reduce treatment during and no deterioration stage of quality of life score which lead to better satisfactory compare to orthodontic-first group. However, some of limitations we need take caution. In future we still need conduct more study to assess the influence of surgery-first method on quality of life.

## Background

Dentofacial deformities are deformities that affect primarily the jaws and the dentition; therefore, they are extremely prominent, are not easily disguised, and affect one’s quality of life immensely [1, 2]. Orthognathic surgery aims to correct these deformities via various osteotomies to achieve a desirable end result. Results deemed to be satisfactory from a clinician’s aspect may not be so from the patient’s aspect, because studies have shown that in patients with facial deformity, there is a close relationship between patient satisfaction and psychosocial functioning [3–6]. In recent years, research on quality of life assessment has been on the rise, and, more importantly, the area of focus has widened with greater emphasis placed on social well-being rather than disease mortality, tumor growth, etc., providing much-neglected subjective views of the treatment outcomes [7]. With increased relevance of health-related quality of life (HRQoL), it is now recognized that quality of life (QoL) assessment is a key outcome measure in the management of dentofacial deformities [8–11].

However, the traditional orthognathic surgery treatment which included preoperative orthodontic treatment, orthognathic surgery, and postoperative orthodontic treatment have several disadvantage, such as long treatment during, worsen facial profile and dental function before received orthognathic surgery. Recently, a new surgery method was introduced to overcome the disadvantages of conventional surgical-orthodontic treatment procedures named surgery-first approach (SFA). Because this surgery did not need pre-operative orthodontic treatment, it can significant reduce total treatment time and improve facial profile immediately which may contribute to improving the satisfaction and quality of life in orthognathic surgerypatients [12–15].

Previous studies had showed that orthognathic surgery could improve dental esthetic and quality of life after treatment [16–18]. A recently systematic review [19] also showed that orthognathic surgery had a positive impact on the patient’s facial appearance and oral function and found social advantages such as improved self confidence. However, the previous studies were concerning the changes of quality of life after traditional surgical-orthodontic treatment, it is also necessary to assess the influence of different surgery time on the quality of life. In additional, there was little research to investigate the improving patients’ satisfaction after orthognathic surgery.

Thus, the aim of this study was to evaluate the changes of oral health related quality of life and satisfaction between surgery-first and orthodontic-first orthognathic surgery patients.

## Methods

### Ethical

Ethical approval was obtained from the Ethics Committee of the stomatology hospital of the WenZhou Medical University. (Ethics approval number: 201506352541) The participants were informed about the trail and given written consent.

### Participants

A total of 50 patients who will receive orthognathic surgery at the Department of Orthodontic at the Stomatology Hospital of WenZhou Medical University were included in this study. The sample divided into 2 groups: the surgery-first group (female 12 , male13; 24.2 ± 5.8 years) and the orthodontic-first group (female 13, male 12; 25.2 ± 4.2 years).

The inclusion criteria were as follows: 1) severe Class III malocclusion need received orthognathic treatment; 2) patients receive either surgery-first or orthodontic-first orthognathic treatment plan;3) orthognathic surgery consisted purely of bilateral sagittal split ramus osteotomy (BSSRO) to resolve mandibular setback. Subjects were excluded from the study if they had cleft lip and/or palate or other craniofacial anomaly; were taking medications; or had undergone previous orthognathic treatment.

Orthodontic treatment was carried out by one clinicians, and the surgical procedures also by one maxillofacial surgeons who familiar with orthodontist.

### Instruments and measures

The patients were given two questionnaires, the Dental Impact on Daily Living (DIDL) questionnaire for assessment of his/her satisfaction after treatment and OHIP-14 for assessment of patient’s quality of life.

During the interviews, we collected the baseline information of patients such as: gender, age, and social-economic status before treatment. For assessing the patient’s satisfaction after treatment, the DIDL questionnaire has 36 items that are placed in five major categories and tackles five major dimensions of dental satisfaction, namely appearance, pain, oral comfort, general performance, and chewing and eating (Appendix 1), was used. The DIDL scale measures the effect and the proportional importance of each dimension to the patient. The scale has a score from 0 to 10 to show the relative importance of each dimension to the patients [20].

The Chinese version of the OHIP-14 which has shown good psychometric properties was used to assess the changes of oral health-related quality of life. The Chinese Version of the OHIP-14 including seven domains: functional limitation, physical pain, psychological discomfort, physical disability, psychological disability, social disability, and any handicaps. Each item was scored on a 5-point scale to rate the OHQOL. The higher scores indicating poorer OHQoL.

The subjects completed six sets of interviews and clinical evaluations at before treatment (T1); 1 month after surgery (T2); 6 months after treatment (T3); 12 months after treatment (T4); and 18 month after treatment (T5); the finished treatment (T6). The pre and post surgical orthodontic period was also recorded.

### Data analysis

Chi square tests were used to compare categorical variables. Repeated-measures analysis of variance (ANOVA) was performed to statistically compare the measurements at each time to the baseline values (T1) in each group. ANOVA determined whether there were any significant overall differences among the groups at each time. Additionally, to identify the significance in each pair of groups, multiple comparison analysis was also performed for the time when the significant difference was noticed. One-way ANOVA and multiple comparison analysis were performed to compare the amount of treatment during between two groups.

All analyses were carried out used Stata software (version 11.2; StataCorp, College Station, Tex). Significance levels were established at 0 .05.

## Results

There was no significant difference between baseline between the surgery-first group and orthodontic-first group, see Table 1.

**Table 1:** Demographic and clinical characteristics of surgery-first group and orthodontic-first group subjects

	Surgery-first group	Orthodontic-first group	*P* value
	n (mean ± SD or %)	n (mean ± SD or %)	
Sex			
Female	12(48)	13(52)	
Male	13(52)	12(48)	NS
Age			
18–25	5	4	
25–30	15	15	
30–35	5	6	NS
Social-economic class			
(high)	20(51)	19(49)	
(low)	5(45)	6(55)	NS

*NS* mean no significant

The mean treatment time was 16.6 + 2.4 month in surgery-first group and 25.3 + 2.4 month in orthodontic-first group. Treatment time was significantly longer in the orthodontic-first group compared to the surgery-first group.

Satisfaction with the DIDL questionnaire was shown in Table 2. There was no significant difference between two groups in each domain. However, the scores were relatively lower in surgery-first group than orthodontic-first group, though this difference did not reach a significant level. This result indicated that surgery-first may acquire better satisfaction compared to orthodontic-first group.

**Table 2:** Frequency of Individual Satisfaction Dimensions in the Study Population

Dimension	Surgery-first group			Orthodontic-first group			P
	Dissatisfied	Relatively Satisfied	Satisfied	Dissatisfied	Relatively Satisfied	Satisfied	
Appearance	0(%)	1(4)	24(96)	0	2(8)	23(92)	NS
Pain	1(4)	7(28)	17(68)	2(8)	6(24)	14(68)	NS
Oral comfort	3(12)	4(16)	18(72)	2(8)	7(28)	16(64)	NS
General performance	0(0)	2(8)	23(92)	0(0)	3(12)	22(88)	NS
Eating and chewing	1(4)	2(8)	22(88)	2(8)	2(8)	21(84)	NS
Total	1(4)	4(16)	20(80)	2(8)	5(20)	18(72)	NS

*NS* Non-significant

Quality of life level in surgery-first group was significantly improved after 1 month surgery (T2) when compared with baseline (*P* < 0.000). However, no statistical differences were observed between T4 (after 12 month ) and T6 (end of treatment). In the orthodontic-first group, the quality of life was deteriorated until T3 (after 6 month treatment) though the difference was not significant. Following orthognathic surgery, a signifcant improved of quality of life levels was observed (*P* < 0.05). The changes in the quality of life levels are shown in detail in Tables 3 and 4.

**Table 3:** Comparison of quality of life scores at before treatment (T1); 1 month after surgery (T2); 6 months after treatment (T3); 12 months after treatment (T4); and 18 month after treatment (T5); the finished treatment(T6) with T1 (before treatment) in two group

Varies	Group	T1	T2	P (T2-T1)	T3	P (T3-T1)	T4	P (T4-T1)	T5	P (T5-T1)	T6	P (T6-T1)
functional limitation	Surgery-first group	8.42	5.22	0.034*	2.39	0.000***	1.99	0.000***	1.98	0.000***	1.89	0.000***
		(2.79)	(1.88)		(1.24)		(1.76)		(1.68)		(1.26)	
	Orthodontic-first group	8.45	8.30	0.867	8.79	0.758	5.42	0.000***	1.98	0.000***	1.92	0.000***
		(2.99)	(2.56)		(1.23)		(1.98)		(1.68)		(1.56)	
physical pain	Surgery-first group	5.26	3.22	0.045*	2.34	0.009**	1.59	0.000***	1.56	0.000***	1.34	0.000***
		(1.85)	(1.55)		(1.24)		(0.67)		(0.51)		(0.89)	
	Orthodontic-first group	5.46	5.30	0.920	6.02	0.052	3.51	0.000***	1.89	0.000***	1.75	0.000***
		(1.61)	(1.69)		(2.36)		(1.72)		(1.28)		(1.22)	
psychological discomfort	Surgery-first group	6.12	4.29	0.048*	2.22	0.000***	0.56	0.000***	0.52	0.000***	0.45	0.000***
		(2.12)	(1.22)		(1.12)		(1.10)		(0.18)		(0.13)	
	Orthodontic-first group	6.25	6.52	0.685	6.56	0.658	4.82	0.000***	2.82	0.000***	0.92	0.000***
		(2.09)	(2.23)		(2.85)		(2.29)		(1.31)		(1.26)	
physical disability	Surgery-first group	6.29	4.15	0.045*	1.64	0.000***	1.34	0.000***	0.12	0.000***	0.10	0.000***
		(2.64)	(1.72)		(1.08)		(1.22)		(0.11)		(0.03)	
	Orthodontic-first group	6.39	6.56	0.687	6.85	0.785	4.42	0.000***	2.41	0.000***	1.49 (0.91)	0.000***
		(2.72)	(2.67)		(2.75)		(1.75)		(1.13)			
psychological disability	Surgery-first group	6.54	3.28	0.007**	1.64	0.000** *	0.42	0.000***	0.20	0.000***	0.15	0.000***
		(2.52)	(1.76)		(1.08)		(0.21)		(0.12)		(0.12)	
	Orthodontic-first group	6.72	6.79	0.920	6.76	0.925	3.72	0.000***	1.83	0.000***	0.88	0.000***
		(2.42)	(3.41)		(3.06)		(1.58)		(1.15)		(1.13)	
social disability	Surgery-first group	6.03	4.26	0.042	1.24	0.000***	0.62	0.000***	0.19	0.000***	0.15	0.000***
		(2.32)	(1.98)		(1.08)		(0.35)		(0.22)		(0.15)	
	Orthodontic-first group	6.12	6.86	0.865	6.89	0.675	4.52	0.000***	1.64	0.000***	0.98	0.000***
		(2.68)	(2.88)		(2.29)		(2.56)		(1.18)		(1.12)	
handicaps	Surgery-first group	3.24	2.18	0.035	1.52	0.004**	0.38	0.000***	0.16	0.000***	0.12	0.000***
		(1.18)	(1.81)		(1.75)		(0.26)		(0.12)		(0.12)	
	Orthodontic-first group	5.66	6.34	0.901	6.61	0.921	2.42	0.000***	1.94	0.000***	0.78	0.000***
		(2.81)	(1.85)		(2.72)		(2.12)		(1.36)		(1.12)	
Total scores	Surgery-first group	38.68	27.72	0.031*	13.94	0.000***	6.90	0.000***	4.11	0.000***	3.89	0.000***
		(4.35)	(3.26)		(2.13)		(1.39)		(0.49)		(1.02)	
	Orthodontic-first group	39.55	41.67	0.654	48.48	0.124	28.86	0.000***	15.61	0.000***	8.68	0.000***
		(4.15)	(4.14)		(3.91)		(3.83)		(2.49)		(1.65)	

*P < 0.05; **P < 0.01; ***P < 0.001

**Table 4:** Statistical evaluation of quality of life scores between the surgery-first group and the orthodontic-first group at 1 month after surgery (T2); 6 months after treatment (T3); 12 months after treatment (T4); and 18 month after treatment (T5); the finished treatment (T6)

Varies	Varies	P(T2-T3)	P(T2-T4)	P(T2-T5)	P(T2-T6)	P(T3-T4)	P(T3-T5)	P(T3-T6)	P(T4-T5)	P(T4-T6)	P(T5-T6)
functional limitation	Surgery-first group	0.024*	0.000***	0.000***	0.000***	0.658	0.725	0.687	0.801	0.831	0.882
	Orthodontic-first group	0.824	0.000***	0.000***	0.000***	0.000***	0.000***	0.000***	0.654	0.702	0.783
physical pain	Surgery-first group	0.048*	0.004**	0.000***	0.000***	0.624	0.742	0.049*	0.903	0.921	0.920
	Orthodontic-first group	0.768	0.000***	0.000***	0.000***	0.000***	0.000***	0.000***	0.726	0.824	0.931
psychological discomfort	Surgery-first group	0.003**	0.000***	0.000***	0.000***	0.000***	0.000***	0.000***	0.869	0.902	0.965
	Orthodontic-first group	0.857	0.000***	0.000***	0.000***	0.000***	0.000***	0.000***	0.867	0.798	0.804
physical disability	Surgery-first group	0.003**	0.000***	0.000***	0.000***	0.687	0.000***	0.000***	0.145	0.263	0.687
	Orthodontic-first group	0.867	0.000***	0.000***	0.000***	0.000***	0.000***	0.000***	0.042*	0.032*	0.903
psychological disability	Surgery-first group	0.004**	0.000***	0.000***	0.000***	0.000***	0.000***	0.000***	0.035*	0.038*	0.867
	Orthodontic-first group	0.864	0.000***	0.000***	0.000***	0.000***	0.000***	0.000***	0.024*	0.034*	0.902
social disability	Surgery-first group	0.000***	0.000***	0.000***	0.000***	0.214	0.000***	0.000***	0.042*	0.038*	0.824
	Orthodontic-first group	0.875	0.000***	0.000***	0.000***	0.000***	0.000***	0.000***	0.042*	0.024*	0.897
handicaps	Surgery-first group	0.321	0.000***	0.000***	0.000***	0.000***	0.000***	0.000***	0.365	0.487	0.867
	Orthodontic-first group	0.806	0.000***	0.000***	0.000***	0.000***	0.000***	0.000***	0.035*	0.008**	0.421
Total scores	Surgery-first group	0.000***	0.000***	0.000***	0.000***	0.000***	0.000***	0.000***	0.042*	0.031*	0.682
	Orthodontic-first group	0.654	0.000***	0.000***	0.000***	0.000***	0.000***	0.000***	0.000***	0.000***	0.725

*P < 0.05; ** *P* < 0.01; *** *P* < 0.001

Though the quality of life scores were lower in surgery-first group, the difference did not come to a significant level between two groups at 1 month, 6 month and 12 month after received orhognhic surgery. The group comparisons are shown in Table 5.

**Table 5:** Comparisons of quality of life scores at the different time points for the surgery-first group and orthodontic-first group at 1 month, 6 month and 12 month after orthognathic surgery

Varies	Postoperative		P	Postoperative		P	Postoperative		P
	1 month			6 month			12 month		
	Surgery-first group(T2)	Orthodontic-first group(T4)		Surgery-first group(T3)	Orthodontic-first group(T5)		Surgery-first group(T4)	Orthodontic-first group(T6)	
functional limitation	5.22	5.42	NS	2.39	1.98	NS	1.99	1.92	NS
	(1.88)	(1.98)		(1.24)	(1.68)		(1.76)	(1.56)	
physical pain	3.22	3.51	NS	2.34	1.89	NS	1.59	1.75	NS
	(1.55)	(1.72)		(1.24)	(1.28)		(0.67)	(1.22)	
psychological discomfort	4.29	4.82	NS	2.22	2.82	NS	0.56	0.92	NS
	(1.22)	(2.29)		(1.12)	(1.31)		(1.10)	(1.26)	
physical disability	4.15	4.42	NS	1.64	2.41	NS	1.34	1.49	NS
	(1.72)	(1.75)		(1.08)	(1.13)		(1.22)	(0.91)	
psychological disability	3.28	3.72	NS	1.64	1.83	NS	0.42	0.88	NS
	(1.76)	(1.58)		(1.08)	(1.15)		(0.21)	(1.13)	
social disability	4.26	4.52	NS	1.24	1.64	NS	0.62	0.98	NS
	(1.98)	(2.56)		(1.08)	(1.18)		(0.35)	(1.12)	
handicaps	2.18	2.42	NS	1.52	1.94	NS	0.38	0.78	NS
	(1.81)	(2.12)		(1.75)	(1.36)		(0.26)	(1.12)	
Total scores	27.72	28.86	NS	13.94	15.61	NS	6.90	8.68	NS
	(3.26)	(3.83)		(2.13)	(2.49)		(1.39)	(1.65)	

*NS* Non-significant

## Discussion

This is the first study to evaluate patient’s satisfaction, treatment duration, and quality of life changes between SFA and conventional orthognathic method in Chinese, and we found some interesting results.

One of the most highlighted benefits of SFA is the reduction in treatment duration. The reason may be that, after surgery, orthodontic tooth movement can be easily achieved because the teeth are usually not occluded. Recently, a systematic review evaluated a few retrospective cohort studies and a larger number of case reports treated with SFA [21]. The results showed that the majority of cases were treated under a year. This agrees with our findings which show an average treatment duration of 10.5 months. This is a clear advantage over the conventional approach where treatment times have been reported in the realm of 7–12 months [22–29]. The different total treatment times for SFA depend on the severity of individual dento-skeletal problems,techniques of surgery, orthodontic mechanics, cooperation and biological response as well as desired results for each patient.

Assessment of patient satisfaction with their dentition after orthodontic treatment was carried out using the DIDL questionnaire. The DIDL questionnaire is a reliable, valid, and comprehensive test to measure patient satisfaction and effect of dental disease on patient daily living [30, 31]. The test has shown the ability to assess satisfaction with different aspects of oral cavity and dental status, and for these reasons, it was selected for this study. Orthodontic problems can affect many aspects of dental esthetics and function, and these aspects are well covered by the DIDL test.

Our results revealed that satisfaction after orthognathic surgery was high.

A total of 80 and 72 % of all patients rated postoperative outcomes after surgery in two group, respectively. There was no significant difference between two groups in each domain. However, the scores were relatively lower in surgery-first group than orthodontic-first group, though this difference did not reach a significant level. This result indicated that surgery-first may acquire better satisfaction compared to orthodontic-first group. The high patient satisfaction in SFA group may due to immediate improvement of facial profile at the beginning of the treatment14,29-30. The high satisfaction rate was in accordance with that reported in previous studies, which ranged between 70 % [32] and 87 % [33], and which was also higher than that reported among pre-treatment and no-treatment control groups [34]. Hence, positive changes occurred in the personality profiles of patients. There was an obvious improvement in self-confidence in 67.5 % of patients as a result of an improved appearance and an improved chewing function.

The results of the present study showed a highly significant degree of overall improvement in patients’quality of life after orthognathic surgery in two group, others found the similar resluts [35–37]. However, we noticed that the quality of life scores was deteriorated before orthognathic in orthodontic-first group. This results indicated that the dental decompensation during pre-orthodontics could worsen the facial deformity, which has been perceived as the most stressful period of overall treatment by the patients [38] Esperao [39] found the similar results. It reminded us that we may need tell patients that he may experience short-term quality of life deterioration before orthognathic surgery.

We were very pleased to find that all the domain of quality of life scores were consistently lower in the surgery-first groups compared to the orthodontic-first group, although there were no significant statistical differences when patients received orthognathic surgery. The reason may be that surgery -first treatment could improve OHRQoL immediately and lead to better satisfactory compare to orthodontic-first group. It was important to doctors, because we not only gain perfect treatment results but also improve patients’quality of life and satisfaction.

The limitations of the study was small sample,we only included 50 patients which may weak the evidence of this study. Secondly, we did not objectively quantify the quality of orthodontic outcome. Although patient satisfaction is high, it has been noted by the orthodontists treating some of these patients that there is an urge to remove the orthodontic appliances very soon after orthognathic surgery. In future research we need including treatment results when assess the quality of life changes. Additionally, because of inclusion criteria of this article was limited to BSSRO, we think if two-jaw surgery was included in the study, the results will be different. In present, because of raised patient's requests, two-jaw surgery increases. Thus, future research need includ more two-jaw surgery patients.

## Conclusions

Surgery-first treatment could significant reduce treatment during and no deterioration stage of quality of life score which lead to better satisfactory compare to orthodontic-first group. However, some of limitations we need take caution, such as small sample, no two-jaw surgery patients were included. In future we need conduct a more larger sample randomized control trail to investigate the oral health-related quality of life in Chinese orthognathic surgery patients.
